# dificultades encontradas durante la pandemia y reportadas por odontólogos

**DOI:** 10.21142/2523-2754-1001-2022-095

**Published:** 2022-03-30

**Authors:** Sonia Brítez Distéfano, Fátima Bañuelos-Gómez, Clarisse Díaz-Reissner, Cynthia Mireya Jara

**Affiliations:** 1 Facultad de Odontologia, Universidad Nacional de Asuncion. Asuncion, Paraguay. sonia_odonto@hotmail.com, fatibanuelos@gmail.com, diazclarisse@gmail.com, cynthiajaraa@gmail.com Universidad Nacional de Asunción Facultad de Odontologia Universidad Nacional de Asuncion Asuncion Paraguay sonia_odonto@hotmail.com fatibanuelos@gmail.com diazclarisse@gmail.com cynthiajaraa@gmail.com; 2 Servicio de Odontologiadel Hospital Central de Policia Rigoberto Caballero. Asuncion, Paraguay. Servicio de Odontologia Hospital Central de Policia Rigoberto Caballero Asuncion Paraguay

**Keywords:** COVID-19, infección por SARS-CoV-2, bioseguridad, salud pública, economía en odontología, COVID-19, SARS-CoV-2 infection, biosafety, public health, dentistry economics

## Abstract

**Objetivo:**

Evaluar las repercusiones económicas y psicológicas, entre otras dificultades encontradas durante la pandemia de COVID-19 por un grupo de odontólogos.

**Materiales y métodos:**

Estudio observacional descriptivo de corte transversal. Se aplicó un cuestionario en línea a odontólogos del Hospital Central de Policía “Rigoberto Caballero” (HCPRC) de Asunción, Paraguay, que fue distribuido entre julio y setiembre del 2021. Constó de 32 preguntas cerradas distribuidas en 5 secciones: datos sociodemográficos, efectos económicos de la pandemia, efectos psicológicos, contagio y experiencias con respecto a la clínica. Los datos fueron analizados con el programa Epi Info versión 7 y los resultados están representados en forma de tablas. También fueron utilizadas la prueba exacta de Fisher y la de chi-cuadrado.

**Resultados:**

Participaron 78 odontólogos, el 78,2% del sexo femenino, el 56,4% de la franja de 35 a 45 años. El 88,5% reportó una disminución de los ingresos en práctica privada y la mayoría tuvo que recurrir a otra fuente para cubrir los gastos diarios. La mayoría relató haber sufrido los síntomas de ansiedad y depresión durante la pandemia, y sintió la necesidad de acudir a un psiquiatra. Reportaron un aumento en la demanda de equipo de protección personal (EPP) y un incremento de sus costos. Casi la mitad de los odontólogos reportaron haberse contagiado de COVID-19.

**Conclusión:**

La pandemia produjo un impacto considerable en los odontólogos del HCPRC, al ocasionar problemas financieros, repercusiones psicológicas y dificultades con los precios y escasez de los EPP.

## Abstract (en)

**Objective:**

To evaluate the economic and psychological repercussions, among other difficulties encountered during the COVID-19 pandemic in a group of dentists.

**Materials and methods:**

Cross-sectional descriptive observational study. An online questionnaire was applied to dentists from the “Rigoberto Caballero” Central Police Hospital (HCPRC) of Asunción-Paraguay that was distributed between July and September 2021. It consisted of 32 closed questions distributed in 5 sections: sociodemographic data, economic effects of the pandemic, psychological effects, contagion, and experiences with respect to the clinic. The data were analyzed with the Epi Info version 7 program and the results are represented in tables, the Fisher's exact test and the Chi-square test were also used.

**Results:**

78 dentists participated, 78.2% female, 56.4% from the 35-45 age group. 88.5% reported a decrease in income in private practice and the majority had to resort to another source to be able to cover daily expenses. Most reported having suffered symptoms of anxiety and depression during the pandemic and felt the need to consult a psychiatrist. It was reported an increase in the demand for Personal Protective Equipment (PPE) and an increase in its costs. Almost half of all dentists reported getting COVID-19.

**Conclusion:**

The pandemic had a considerable impact on HCPRC dentists, causing financial problems, psychological repercussions and difficulties with prices and a shortage of PPE.

## INTRODUCCIÓN

El coronavirus ya ha afectado a casi 300 millones de personas en todo el mundo ^1^. Las evidencias sugieren que el contagio se produce principalmente por medio de gotitas, estornudos y aerosoles ^2^, y que existe un alto riesgo para el profesional odontólogo por la constante exposición producida en la mayoría de los procedimientos dentales que utilizan alta rotación ^3^. Actualmente, son muchos los odontólogos que se encuentran preocupados por contraer COVID -19 en su lugar de trabajo ^4^. Por esto, durante la pandemia, especialmente en la fase de cuarentena, muchos de ellos han disminuido sus horas laborales en las clínicas dentales; asimismo, se vieron limitados a realizar solo tratamientos de urgencia con el fin de mitigar el riesgo de transmisión del virus ^5^. Indudablemente, estas medidas han impactado negativamente en la economía ^6^ y podrían seguir repercutiendo en el futuro ^7^. Se ha reportado una pérdida importante en los ingresos, inclusive después de la fase de la cuarentena estricta, lo que ha generado preocupación en los odontólogos acerca de los futuros efectos de la pandemia en la profesión ^8^. 

A nivel mundial, son muchos los trabajadores de blanco que se encuentran en un estado de ansiedad y miedo, con los cambios repentinos en los protocolos de atención, además de las diversas adecuaciones de los consultorios a las pautas recomendadas en cada país ^9^. Se ha evidenciado una alta prevalencia de depresión y ansiedad en los odontólogos ^10^, y ha sido más elevado el estrés en mujeres que en hombres ^11^, con niveles de ansiedad moderada en la mayoría de los casos ^12^. Además, fue reportado un mayor impacto psicológico en aquellos que trabajan en el sector privado, en comparación con los del sector público ^10^.

Un estudio reciente realizado en Paraguay ^13^ evaluó los protocolos de control de infecciones utilizados por los odontólogos en ese país, así como el porcentaje de contagios después de atender a pacientes positivos a la COVID-19. Sin embargo, no fueron abordadas las dificultades encontradas en la práctica clínica ni las repercusiones psicológicas y económicas de la pandemia. Así, el presente estudio tuvo como objetivo evaluar las repercusiones económicas y psicológicas, entre otras dificultades encontradas durante la pandemia de COVID-19 en odontólogos de Asunción (Paraguay).

## MATERIALES Y MÉTODOS

El estudio es observacional descriptivo de corte transversal. Se aplicó un cuestionario a odontólogos del Hospital Central de Policías Rigoberto Caballero (HCPRC), de Asunción, Paraguay. Se incluyó a odontólogos sin límite de edad y fueron excluidos aquellos sin acceso a internet. La selección de los participantes se realizó utilizando la lista de odontólogos que prestan servicio en dicho hospital, proporcionada por el Departamento de Recursos Humanos de la institución, con sus respectivos contactos. La encuesta fue anónima y voluntaria. Al abrir el enlace, el título y los objetivos del trabajo eran presentados, así como los beneficios y riesgos de participar en el mismo. Este trabajo contó con la aprobación de la Dirección de Docencia e Investigación de la Sanidad Policial (N.o Expdte. 2128). 

El cuestionario en línea (Google Forms) se estructuró utilizando como modelo un trabajo previamente publicado en Irán ^5^, con 32 preguntas cerradas distribuidas en 5 secciones de la siguiente manera: datos sociodemográficos, efectos económicos de la pandemia, efectos psicológicos, contagio y experiencias con respecto a la clínica. El reclutamiento de los participantes se realizó a través de una invitación personalizada, que se envió como mensaje de WhatsApp entre julio y setiembre del 2021. El tamaño de muestra referencial fue calculado teniendo en cuenta un estudio previo iraní ^5^ en el cual el 51% de los encuestados relataron haber tenido síntomas de ansiedad y depresión durante la pandemia, con un nivel de confianza del 95% y una precisión del 5%. Fue necesario un mínimo de 71 participantes, pero al considerar una posible pérdida del 10%, se requirió de 78 encuestados. El muestreo fue no probabilístico por conveniencia. 

Los datos fueron analizados con el programa Epi Info versión 7 y los resultados están representados en tablas. Para el análisis de las variables “disminución de ingresos” (sí/no) y “síntomas de depresión” (acuerdo/desacuerdo) se utilizó la prueba exacta de Fisher, mientras que para “problemas financieros” (sí/no) y “síntomas de depresión” (acuerdo/desacuerdo) se utilizó la prueba de chi-cuadrado con un nivel de confianza del 95%.

## RESULTADOS

Respondieron a la encuesta 78 odontólogos, el 78,2% del sexo femenino. Se agruparon en tres franjas etarias: 24-34 años (21,8%), 35-45 (56,4%) y 46 o más (21,8%). Los años de experiencia fueron clasificados en <5 años (15,4%), 5-10 (15,4%), 11-20 (38,5%) y ≥21 años (30,8%). El campo clínico de mayor práctica (se podía marcar más de una opción) fue la odontología general, con un 64,1%, seguido por la ortodoncia (20,5%), la odontopediatría (9,0%), la endodoncia (9,0%), la cirugía (6,4%) y la cirugía maxilofacial (2,6%). 

El 74,4% declaró que, además de prestar servicio en el HCPRC, ejercen la profesión en clínicas privadas; el 37,2% trabaja en otro servicio público; el 12,8% se dedica a la docencia y el 3,9% trabaja para un seguro odontológico.

Con respecto a las dificultades económicas, el 88,5% reportó una disminución de los ingresos en su práctica privada y la mayoría tuvo que recurrir a otra fuente para cubrir los gastos diarios. Un 24,4% pagó el salario de los asistentes dentales independientemente del cierre de las clínicas (Tabla 1). 

**Tabla 1:** Distribución de odontólogos según dificultades económicas debido a la pandemia (n = 78)

Dificultades económicas durante la pandemia	Respuestas				Total
	Sí	No	No aplica	Prefiero no declarar	
Una disminución de los ingresos	88,5%	11,5%	0,0%	0,0%	100,0%
Usó otra fuente de ingresos (fuera de la práctica dental) para gastos diarios	61,5%	38,5%	0,0%	0,0%	100,0%
Continuará con problemas financieros en el futuro	46,2%	53,9%	0,0%	0,0%	100,0%
Tuvo que pagar el salario de los asistentes independientemente del cierre de las clínicas dentales	24,4%	10,3%	65,4%	0,0%	100,0%
No cambié mis horas de trabajo y realicé procedimientos que no son de emergencia debido a razones financieras	37,2%	53,9%	0,0%	9,0%	100,0%
Despidió a los asistentes por problemas económicos	11,5%	32,1%	56,4%	0,0%	100,0%
Se ha incrementado el uso de EPP	97,4%	2,6%	0,0%	0,0%	100,0%
Aumento del precio de los EPP	92,3%	7,7%	0,0%	0,0%	100,0%
Recibió ayuda de organizaciones públicas para proporcionar EPP	10,3%	89,7%	0,0%	0,0%	100,0%
Los asistentes decidieron no trabajar durante la pandemia	9,0%	26,9%	64,1%	0,0%	100,0%
Recibió ayuda financiera de organizaciones públicas	7,7%	92,3%	0,0%	0,0%	100,0%

Con respecto a los efectos psicológicos, la mayoría relató haber sufrido los síntomas de ansiedad y depresión durante la pandemia, y sintieron la necesidad de acudir a un psiquiatra (Tabla 2).

**Tabla 2:** Distribución de odontólogos según el nivel de acuerdo sobre efectos psicológicos de la pandemia (n = 78)

Percepción sobre los efectos psicológicos de la pandemia	Nivel de acuerdo					Total
	Totalmente de acuerdo	De acuerdo	Algo de acuerdo	En desacuerdo	Prefiero no declarar	
Tuve síntomas de ansiedad y depresión durante estos tiempos	20,5%	32,1%	16,7%	30,8%	0,0%	100,0%
Seguir las últimas noticias me provoca depresión y ansiedad	18,0%	30,8%	42,3%	9,0%	0,0%	100,0%
Siento que necesito consultar con un psiquiatra	9,0%	15,4%	66,7%	0,0%	9,0%	100,0%

La disminución de ingresos no se asoció significativamente con la presencia de síntomas de depresión (p = 0,45). Por otro lado, los participantes que relataron tener síntomas de depresión presentaron una visión significativamente (p = 0,04) más pesimista con respecto a su futuro económico, relatando que probablemente continuarían con problemas financieros a largo plazo. 

El 47,4% de los encuestados mencionó haber padecido síntomas relacionados con la COVID-19; entre ellos, el 42,3% dio positivo a la prueba. El 23,1% de los asistentes dentales de los entrevistados también tuvo prueba de COVID-19 positiva (Tabla 3).

**Tabla 3:** Distribución de odontólogos según preguntas sobre síntomas de la COVID-19 (n = 78)

Síntomas de la COVID-19		Frecuencia	Porcentaje
¿Ha visitado pacientes de alto riesgo?	Sí	18	23,1%
	No	60	76,9%
¿Tuvo síntomas de COVID-19?	Sí	37	47,4%
	No	41	52,6%
¿Tuvo una prueba positiva para COVID-19?	Sí	33	42,3%
	No	45	57,7%
¿Su asistente tuvo síntomas de COVID-19?	Sí	17	21,8%
	No	24	30,8%
	No tengo asistente	35	44,9%
	Prefiero no declarar	2	2,6%
¿Su asistente dio positivo en la prueba de COVID-19?	Sí	18	23,1%
	No	23	29,5%
	No cuento con asistente	35	44,9%
	Prefiero no declarar	2	2,6%
Total		78	100,0%

Entre las principales modificaciones durante la atención en el consultorio, el 57,7% de los odontólogos mencionó que solo realiza tratamientos de emergencia, urgencia o impostergables que puedan derivar en urgencia. Más de la mitad opina que, luego del final de la fase de alerta, ya podría ser momento para reactivar la atención clínica (Tabla 4).

**Tabla 4:** Experiencia de los odontólogos durante la pandemia (n = 78)

Preguntas		Frec	%
Seguí las últimas pautas respecto de la práctica dental durante la pandemia	Sí	73	93,6
	No	3	3,9
	Prefiero no declarar	2	2,6
Cambio en sus planes de tratamiento durante la pandemia	Solo realizo tratamientos de emergencia, urgencia o impostergables	45	57,7
	Cancelado todo el tratamiento hasta el final de la fase de alerta de la pandemia	16	20,5
	Nada ha cambiado	12	15,4
	Cancelados todos los tratamientos hasta el final de la pandemia	5	6,4
¿Hubo aumento de llamadas de pacientes?	Sí	53	68,0
	No	25	32,1
Momento en que deben reactivarse las clínicas	Luego del final de la fase de alerta	48	61,5
	Luego del final de la pandemia de COVID-19	15	19,2
	Las clínicas deberían estar abiertas ahoras	15	19,2
Su estrategia principal ante la reapertura de clínicas dentales	Usando el equipo de protección personal	50	64,1
	Atender pacientes que no tienen síntomas de COVID-19	13	16,7
	Prueba COVID-19 para pacientes previa a la consulta	9	11,5
	Trabajar cuando termine la pandemia de COVID-19	6	7,7
Equipos odontológicos que han sido escasos durante la pandemia	Tapabocas N95	48	61,5
	Guantes quirúrgicos	27	34,6
	Tapabocas quirúrgico	23	29,5
	Protector facial transparente	14	18,0
	Bata impermeable	11	14,1
	Protector ocular	11	14,1
	Bata desechable	9	11,5
	Soluciones desinfectantes	7	9,0
	No he tenido problemas para encontrar EPP	20	25,6
Total		78	100

El sexo no estuvo asociado con un mayor nivel de ansiedad o depresión, ni con un mayor porcentaje de prueba positiva para la COVID-19. Por otro lado, con respecto a la modificación de la rutina dentro del consultorio, las mujeres solo realizaron tratamientos de urgencia en una proporción significativamente mayor que los hombres (Tabla 5). 

**Tabla 5:** Distribución de odontólogos por sexo según haya tenido resultado positivo en la prueba de COVID-19, síntomas de depresión o ansiedad, y haya realizado solo tratamientos de urgencia a sus pacientes (n = 78)

Preguntas		Sexo		Prueba chi-cuadrado p-valor
		Femenino (n = 61)	Masculino (n = 17)	
Prueba positiva COVID-19	Sí	24	9	0,316
		39,3%	52,9%	
	No	37	8	
		60,7%	47,1%	
Síntomas de depresión o ansiedad	Sí	43	11	0,648
		70,5%	64,7%	
	No	18	6	
		29,5%	35,3%	
Solo realizó tratamiento de emergencia	Sí	39	6	0,035*
		63,9%	35,3%	
	No	22	11	
		36,1%	64,7%	

*Estadísticamente significativo

Con respecto al aumento de la consulta telefónica, el 52,6% de los participantes indicó estar “algo de acuerdo” con que fue un método eficaz para resolver los problemas de los pacientes, mientras que el 2,6% y el 20,5% declararon estar “totalmente de acuerdo” y “de acuerdo”, respectivamente. Por otro lado, el 24,4% cree que las teleconsultas son un método poco eficaz para resolver los inconvenientes relatados por los pacientes. 

Al final de la encuesta, se consultó sobre el tipo de actividades realizadas en el HCPRC durante el periodo de pandemia. El servicio del hospital había quedado limitado a atenciones exclusivas de urgencias, y el 100% del personal fue reasignado a otra función. El 79,5% de los odontólogos fue a Recepción, Acogida y Clasificación (RAC) de pacientes, mientras que el resto cumplió las funciones de atención en la central telefónica, laboratorio y archivo.

## DISCUSIÓN

Los resultados del presente estudio son reveladores porque exponen las múltiples dificultades enfrentadas por los odontólogos durante el periodo de pandemia. Fueron reportados efectos psicológicos y económicos, así como el aumento de la demanda de EPP y el incremento de sus costos. Casi la mitad de los odontólogos reportaron haberse contagiado de COVID-19.

Durante la fase de alerta, los servicios odontológicos, tanto públicos como privados, se vieron afectados por la pandemia ^14^. El 100% de los profesionales del HCPRC fueron reasignados a otra función, ya sea al servicio de RAC o la central telefónica, y las atenciones del consultorio se reservaron para casos de urgencia. La gran mayoría de los encuestados ejerce la profesión de manera paralela en un consultorio privado, por lo que fue elevado el porcentaje de odontólogos (88,5%) que señalaron una disminución en sus ingresos pese a contar con el salario fijo brindado por el HCPRC. Menos del 10% recibió ayuda pública para compensar la falta de ingresos en el sector privado y, junto con la baja demanda de pacientes, como consecuencia de las restricciones, un gran número de asistentes dentales fueron despedidos. Las ayudas sociales brindadas por el Gobierno, tales como Tekoporã ^15^ y Ñangareko ^16^, estaban dirigidas a familias en situación de pobreza o a trabajadores informales sin seguro social. 

Casi el 60% redujo sus actividades laborales privadas a la realización exclusiva de tratamientos de urgencias. Las mujeres indicaron lo anterior en una proporción significativamente superior a los hombres. Estos últimos, en mayor medida, dijeron realizar otros tipos de tratamientos. Algunos odontólogos relataron que, por cuestiones financieras, realizaron todo tipo de tratamiento. En un estudio similar ^17^, el 91,29% de los odontólogos suspendió sus actividades clínicas durante la cuarentena y el 77,96% ha pensado en disminuir sus horas de trabajo. Las repercusiones económicas en odontólogos ya fueron reportados en diferentes países. En España ^7^ se encontró que, del total de hombres encuestados, el 33,1% sufrió pérdidas superiores a >15 000 €, frente al 19,4% de las mujeres encuestadas (OR = 3,121, p < 0,001). Sin embargo, a diferencia de Paraguay, casi el 30% de los españoles recibió ayuda del Estado durante el periodo de cuarentena estricta. Experiencias similares a las de Paraguay fueron reportadas por odontólogos de Italia ^8^, donde el 86% de los profesionales informa alguna pérdida de ingresos y el 94% teme una caída en los pacientes después de la fase de cuarentena. La escasez de insumos, entre los que se destacan las mascarillas y los guantes, junto con la subida de los precios por el aumento de la demanda, fueron las dificultades económicas más reportadas. 

Con respecto a las repercusiones psicológicas, casi el 70% de los encuestados señala haber padecido cierto nivel del ansiedad o depresión, y un porcentaje similar cree que debería acudir al psiquiatra. Resultados igual de alarmantes fueron encontrados en un estudio realizado en Italia ^18^ donde, a través de una evaluación psicológica, se pudo observar que el 9% presentó ansiedad severa, mientras que el 85% de los dentistas informó estar preocupado por contraer la infección durante la actividad clínica. Generalmente, la inseguridad laboral y el miedo a contraer COVID-19 se asocian positivamente con los síntomas depresivos ^19^. En el presente estudio, la asociación entre los síntomas de depresión o ansiedad no estuvieron asociadas con el sexo, lo cual difiere de datos previos ^11^, en los que estos síntomas se presentaron en mayor medida en las mujeres. Una mayor angustia psicológica también fue reportada anteriormente entre aquellos que tienen una enfermedad preexistente, miedo a contraer COVID-19 del paciente y una mayor sobrecarga subjetiva (percepciones sobre sus circunstancias que, junto con sus estrategias de afrontamiento, determinan el nivel de su estrés laboral) ^20^.

El porcentaje de contagio entre los odontólogos y asistentes dentales fue alto. En el presente estudio, más del 40% dio positivo para COVID-19, mientras que en un estudio previo publicado en el país ^13^ solo el 13,39% de los odontólogos dio positivo. Es importante mencionar que el período en el que se realizaron ambos estudios fue diferente. En enero del 2021, en el país, habían sido confirmados 132 548 casos ^21^, mientras que este estudio fue realizado en agosto, cuando el número de confirmados ascendía a 457 612 ^22^. 

Se reportó un aumento de las teleconsultas; sin embargo, solo el 2,6% estuvo totalmente de acuerdo con que es un método eficaz para resolver el problema del paciente. La mayoría de los profesionales mencionaron que la principal estrategia utilizada ante la reapertura de las clínicas dentales debería ser la utilización del EPP. Un muy bajo porcentaje de odontólogos consideró efectiva la estrategia de atender pacientes sin síntomas compatibles con la COVID-19. Una reciente revisión sistemática ^23^, que incluye 70 artículos, concluye en que, “según la jerarquía del control de peligros, el EPP debe ser el último recurso para prevenir la propagación de enfermedades infecciosas y solo debe usarse para protección y no para controlar la transmisión”. Por tanto, podría suponerse que los encuestados demostraron interés en protegerse con los elementos de bioseguridad, lo cual podría ser considerado positivo; no obstante, todavía es necesaria una comprensión más profunda de los modos de transmisión de la enfermedad para aplicar medidas de control efectivas que eviten la transmisión del virus. La implementación de un correcto triaje telefónico y el triaje dentro del consultorio son las primeras medidas sugeridas dentro del protocolo de atención divulgado por el Ministerio de Salud Pública y Bienestar Social del Paraguay ^14^.

Algunas limitaciones del estudio deben ser consideradas. La muestra correspondía a odontólogos de un servicio público, los cuales contaron con un salario estable durante toda la pandemia, independientemente de las restricciones en la atención. Como ya fue mencionado, los profesionales fueron reasignados a otros puestos dentro del hospital, a fin de compensar el salario percibido. Todas las repercusiones económicas mencionadas podrían no representar la realidad de los odontólogos dedicados exclusivamente a la atención en el sector privado, por lo que el impacto económico podría estar subestimado en el presente estudio. Otra limitación del cuestionario fue la falta de preguntas sobre la fuente de contagio y la vacunación. El alto porcentaje de odontólogos contagiados no necesariamente implica que estos hayan adquirido la enfermedad durante la atención dental. El porcentaje de vacunados también podría ser un dato interesante para recabar en futuras investigaciones, ya que así podría conocerse el número de profesionales que presentaron prueba positiva para el COVID-19 antes y después de recibir el esquema de vacunación completa. 

El presente estudio visibilizó el impacto que la pandemia produjo sobre los odontólogos del HCPRC, con lo que fue posible acrecentar esfuerzos en cuanto a la prevención. Debido al alto porcentaje de contagio observado, fueron reforzados los protocolos de triaje previos a la consulta; para ello, se solicitó a los pacientes firmar un documento en el que dieran fe de que, en los últimos 14 días, no tuvieron ningún síntoma respiratorio ni contacto con personas positivas a COVID-19, así como no haber viajado a países con alerta epidemiológica. Las atenciones se continuaron llevando a cabo de forma espaciada, para evitar las aglomeraciones tanto en los consultorios como en la sala de espera. El hospital cuenta con atención psicológica gratuita para sus funcionarios, y su utilización ha sido recomendada. 

## CONCLUSIÓN

La pandemia produjo un impacto considerable en los odontólogos del HCPRC, que generó problemas financieros y repercusiones psicológicas. Entre otras dificultades mencionadas se encuentran el incremento del uso de EPP, la escasez y el elevado precio de estos.

## Fuente de financiamiento:

Financiado por los investigadores
